# Baseline predictors of maintenance of intervention-induced changes in physical activity and sitting time among diabetic and pre-diabetic patients: a descriptive case series

**DOI:** 10.1186/1756-0500-6-190

**Published:** 2013-05-08

**Authors:** Judith HM Helmink, Jessica S Gubbels, Femke N van Brussel-Visser, Nanne K de Vries, Stef PJ Kremers

**Affiliations:** 1 Department of Health Promotion, Maastricht University, School for Nutrition, Toxicology and Metabolism (NUTRIM), Maastricht, MD 6200, the Netherlands; 2 Netherlands Institute for Sport and Physical Activity, Ede, the Netherlands; 3 Department of Health Promotion, Maastricht University, School for Public Health and Primary Care (CAPHRI) and School for Nutrition, Toxicology and Metabolism (NUTRIM), Maastrich, the Netherlands

**Keywords:** Lifestyle intervention, Type 2 diabetes, Sitting time, Physical activity, Socio-cognitive profile

## Abstract

**Background:**

The aim of this study was to explore the predictive value of baseline characteristics in relation to changes in physical activity (PA) and sedentary behaviour among diabetic and pre-diabetic patients participating in a primary care based exercise intervention. We used a descriptive case series among diabetic and pre-diabetic patients (n = 119, 50.8% male, mean age 65.5 (SD = 7.8)). Measurements took place with questionnaires at baseline and two years after the start of the intervention. Predictor variables included demographic factors, Body Mass Index, baseline PA and sitting time, and baseline socio-cognitive profile.

**Results:**

At follow-up, respondents spent more time being physically active than at baseline. For the total group, the average sitting time remained almost unchanged between the two measurements. Further exploration showed that respondents who had relatively high levels of PA at the start of the intervention, increased their total sitting time, while respondents with relatively low levels of PA at the start decreased their sitting time. The socio-cognitive profile did not predict behaviour change. The intervention appeared to be suitable for people with a low-education level, but the results should be interpreted in view of the limitations of the study such as the non-controlled design, self-reported outcomes and selective drop-out of participants.

**Conclusions:**

Interventions for this specific target group may need to put more emphasis on the prevention of increased sitting time. The finding that the socio-cognitive profile did not predict behaviour change may underline the proposition that decisions to initiate and maintain PA behaviour change are to a large extend non-linear events. Acknowledging the possible non-linearity of the relationship between socio-cognitive determinants and behaviour change will help our understanding of this complex and dynamic process.

## Background

Type 2 diabetes mellitus is one of the most common chronic diseases worldwide, and its incidence is increasing everywhere [1]. The worldwide prevalence of type 2 diabetes was 171 million in 2000 and is expected to increase to 366 million in 2030 [1]. Benefits to health can be achieved by getting inactive people with diabetes to become more active, which can delay the development of complications in the long term and postpone pharmaceutical treatment [2,3]. Being physically active is not only important in the treatment of type 2 diabetes, but also decreases the risk of developing the disease [4], making it an important preventive measure [5]. Several programmes have been developed to decrease the incidence of type 2 diabetes by targeting the pre-diabetic patient population (i.e. people with an impaired fasting glucose) [2,6-8]. In view of the gap between theory and practice, the challenge is to develop effective primary care exercise programmes. In addition, accumulating evidence indicates the importance of reducing sitting time in addition to increasing physical activity (PA) [9-11]. Therefore, primary care exercise programmes should not only aim to increase total PA time, but also to reduce sedentary behaviour.

A study of five primary care exercise programmes in Denmark found that participants had increased their PA levels after the intervention. The results of this study suggested that between three and six participants have to enrol in a programme for one to achieve an important increase in PA behaviour [12]. A review by Williams [13] indicated that 17 inactive participants had to start an exercise-on-prescription intervention for one to become active at a moderate intensity level [13]. Furthermore, a review by Garrett and colleagues [14] showed that PA interventions through primary care are more cost-effective than intensive gym-based or instructor-led interventions [14]. However, maintaining intervention-induced behavioural changes remains difficult for many people [13].

Research to identify baseline characteristics that predict intervention-induced behaviour change and maintenance is still in its infancy [15]. Intervention studies have typically focused on process indicators of intervention success (e.g. satisfaction, level of use), while others tried to identify mediating cognitive changes (e.g. attitudes, self-efficacy). Although this information is highly relevant for an understanding of the mechanisms of intervention effects, it does not provide health care professionals with information on the responsiveness of particular risk groups to the intervention. Such information would make the efforts of the health professionals more cost-effective, and would provide intervention designers with feedback on risk groups that may not be responsive to the intervention.

The baseline cognitive profile of intervention participants may be of importance in predicting responsiveness to interventions. Three types of determinants are generally distinguished in behavioural theories: outcome expectancies or attitude, subjective norm and self-efficacy. Outcome expectancies refer to a person’s perception that a given behaviour will lead to certain outcomes [16] and attitudes are determined by the beliefs about whether the behaviour will lead to desirable or undesirable outcomes [17]. Subjective norms refer to expectations about what people in the social environment want you to do [18]. Finally, self-efficacy (or perceived behavioural control) reflects a person’s expectation that he or she can indeed perform the desired behaviour [19]. These cognitions have been successfully applied in research on PA (e.g. [20-23]).

Studies have revealed, however, that it is not cognitions but past behaviour that is the most important factor in predicting behaviour change. One study showed that past behaviour was the strongest predictor of behaviour at follow-up [24,25]. A study by Plotnikoff [25] showed that although the Theory of Planned Behaviour (TPB) was useful for designing a PA promotion intervention for diabetes patients, the level of PA at six months after the intervention was best predicted by the level of PA at baseline, rather than by the TPB-based cognitions [25].

Another explanation for the increase or decrease in PA may be weight status. A population-based survey in Australia showed that, over a 5-year period, abdominal obesity predicted the decrease in PA for both men and women [26]. This is in line with the outcomes of a review which found a high body mass index (BMI) to be associated with less PA [27]. The study by Lakerveld also revealed that women who had high scores for sitting time at baseline were more likely to have reduced their PA levels after five years [26]. As regards gender differences, women were found to be less active than men [27]. Furthermore, as the age of respondents increases, the level of PA tends to decrease [27,28]. Lower educational levels were also associated with less PA [27]. On the other hand, being employed was associated with more PA [28].

The aim of this study was to explore the predictive value of baseline characteristics with changes in physical activity and sedentary behaviour among diabetic and pre-diabetic patients participating in a primary care based exercise intervention. Predictor variables included demographic factors, Body Mass Index, baseline PA and sitting time, and baseline socio-cognitive profile. Our main hypothesis was that, corrected for relevant socio-demographic factors, positive characteristics of the socio-cognitive profile (e.g. more positive attitude towards being active) would predict larger changes in physical activity.

## Methods

### Participants and design

Respondents in this study were people who had agreed to participate in a primary care exercise programme called BeweegKuur (see [29] for more information). People with an impaired fasting glucose (fasting glucose value (finger prick) ≥ 5.6 to ≤ 6.0 mmol/L or fasting plasma glucose value ≥ 6.1 to < 6.9 mmol/L) and persons with type 2 diabetes (according to the 2006 definition; HbA1c ≥ 7.0) [30] were eligible for inclusion in the intervention. Exclusion criteria were type 2 diabetes combined with three or more complications, serious poly-pharmacy or type 3 hypertension. The aim of the 12-month intervention is to guide participants with an impaired fasting glucose or type 2 diabetes in achieving a sustained healthy lifestyle [31]. Eligible patients must have an inactive lifestyle (i.e. not meeting the Dutch guideline recommendation of exercising for at least half an hour on five or more days a week) and motivation for behavioural change. The motivation for behavioural changes is subjectively determined by the general practitioner (GP) and lifestyle advisor (LSA). The selection of participants differed between the health care professionals; some selected them during consultation, while others actively approached potential participants.

In the BeweegKuur programme, a patient’s GP determines whether he or she is eligible for the programme, and patients are referred to a LSA, usually the practice nurse. The LSA provides coaching and supervision, based on principles of Motivational Interviewing [32], and designs an individual exercise programme in close collaboration with the patient. In addition, all participants are referred to a dietician. The GP’s practice remains the central location during the BeweegKuur, where patients have frequent contact with the LSA about their progress in the programme and about perceived barriers, with more frequent and intensive appointments during the first three months. The final appointment with the LSA takes place one year after the start of the intervention. After the intervention, participants are expected to go on exercising on their own, using local exercise facilities.

The present study was a descriptive case series, based on the baseline and follow-up measurement data using two questionnaires. The Maastricht University Medical Ethics Committee indicated that its approval was not required for this study. The LSA was responsible for the distribution of the questionnaires to the patients. The participants received the first questionnaire (t = 0) at the start of the BeweegKuur intervention. The recruitment period was nine months. The respondents were exposed to the intervention for one year. The second questionnaire (t = 1) was sent to the LSA two years after the start of the intervention, after a one-year follow-up period without intervention. Patients completed an informed consent form at baseline. Both questionnaires had to be returned to the university free of postage. A gift voucher was raffled among participants. The baseline questionnaire was completed by 361 participants, 29 of whom could not be reached by their LSA for the second questionnaire. In total, the second measurement was completed by 119 respondents (response rate 35.8%). Drop-out analyses showed that the persons who did not complete the second questionnaire did not differ from those who completed both questionnaires, in terms of age, occupation, country of birth, BMI, vigorous-intensity PA time, walking time or total sitting time at baseline. Gender, education, moderate-intensity activity and motivation at baseline were significant predictors of response at the second measurement. Men were more likely to fill in the second questionnaire than women (*p =* 0.04). Respondents with a high (*p* < 0.01) or medium-level (*p* = 0.03) education were more likely to fill in the second questionnaire than low-educated respondents. Respondents with a higher level of moderate-intensity activity at baseline (*p* = 0.04) and with a higher motivation at baseline (*p* = 0.01) were more likely to respond to the second questionnaire.

### Baseline measures

#### Demographic characteristics, weight, PA behaviour and sitting time

The questionnaire used in this study was designed specifically for the current study, based on validated questionnaires and theory. The scorings method of the domains used in the questionnaire to assess the cognitive profile corresponds with recommendations of the primary theoretical frameworks [18,19]. Demographic variables assessed in the questionnaire were date of birth, gender, educational level (low, medium or high) and occupation. Self-reported weight and height were used to calculate BMI (weight (kg) / height (m)^2^). PA and sitting time were measured with the validated short version of the International Physical Activity Questionnaire (IPAQ) [33], which assessed PA time in a usual week in terms of total time spent on walking and on moderate-intensity and vigorous-intensity PA. Total PA time in was measured by calculating the amount of moderate and vigorous PA time. The proxy measure of sedentary behaviour was the time spent sitting on an ordinary weekday. This was measured with the question: ‘During the last 7 days, how much time did you spend sitting on a week day?’, where the respondents could fill in the hours or minutes spent sitting. The sitting time includes time spent at work, at home, while doing course work and during leisure time [33]. Activity data were examined for distribution and outliers. Outliers were identified and recoded to the 95^th^ percentile of the distribution.

#### Socio-cognitive profile

The questionnaire items regarding the socio-cognitive profile addressed outcome expectancies [16], attitude, subjective norm and self-efficacy towards participating in physical activity intervention [18,19]. Outcome expectancies were measured with eleven items, with answers on a 5-point scale (‘I do not agree at all’ (1) to ‘I fully agree’ (5)). Reliability analysis yielded a Cronbach’s alpha of 0.74. An example of an item is ‘If I participate in BeweegKuur, I will lose weight’. The attitude concept was measured on a 5-point scale (‘I do not agree at all’ (1) to ‘I fully agree’ (5)) with six items (α = 0.65), for example: ‘The BeweegKuur programme suits people like me’. Five self-efficacy questions (α = 0.83) were asked, using 5-point scales with answering categories ranging from ‘I do not agree at all’ (1) to ‘I fully agree’ (5). An example of a question is ‘I think I will be able to attend the whole BeweegKuur programme.’ The subjective norm was measured with three questions on 5-point scales (‘I do not agree at all’ (1) to ‘I fully agree’ (5) (α =0.88)). An example of these questions is ´My family wants me to participate in BeweegKuur.’ Motivation was assessed by the question ‘How motivated are you to be more physically active?’, with answering categories on a scale from 0 (not motivated) to 10 (extremely motivated).

### Follow-up measures

The measures that were included to study the maintenance of behavioural changes were PA (vigorous-intensity, moderate-intensity and walking time) and sitting time. The measures were identical to those used in the baseline measurement.

### Statistical analyses

The descriptive statistical analyses were conducted using SPSS 17.0. Frequencies and descriptives were used to assess the background characteristics of the respondents. Shapiro-Wilk tests and sample distribution plots were used to test the normality of the PA measures, total sitting time and BMI at baseline and follow-up, as well as the socio-cognitive profile variables at baseline. All variables were non-normally distributed, except for BMI and outcome expectations. Therefore, non-parametric tests were used to further examine the data. Wilcoxon’s signed-rank tests were performed to analyze differences in PA measures and total sitting at baseline and at the second measurement.

In addition, linear regression analyses were conducted. For this purpose, we calculated the change scores between baseline (T0) and follow-up (T1) for the following outcome variables: total vigorous-intensity PA time, total moderate-intensity PA time, total walking time and total sitting time. These change scores were used as the dependent variables in the regressions, while independent variables were demographic characteristics (gender, age, education and occupation) as well as the baseline data on BMI, socio-cognitive profile, PA (walking, moderate- and vigorous-intensity PA), and total sitting time. Sensitivity analyses were performed to check the robustness of the results. The regression analyses described above where therefore repeated, excluding the respondents below the 5^th^ percentile and above the 95^th^ percentile of the relevant activity change scores.

Potential suppressor or mediation effects of PA behaviour in our multivariate equations were checked for using bivariate Spearman correlations of the socio-cognitive profile factors and all outcomes.

Finally, if statistically significant predictors were found, the responsiveness among respondent groups with certain baseline characteristics was explored by means of a median-split analysis for demographics, weight status (non-obese vs obese), socio-cognitive profile, PA and sitting time. Mann–Whitney tests and graphic representations were used to test and interpret the differences between groups. In all analyses, p-values <0.05 were considered statistically significant.

## Results

Slightly more than half of the respondents were men, and the mean age was 65 years. Almost half of the respondents were low–educated, and 52.2% had a BMI above 30 (see Table 1).

**Table 1:** **Descriptive characteristics of the participants (n = 119)**^**a**^

	**N**	**%**	**Mean ± SD**		**N**	**%**
**Gender**				**Occupation**		
Male	60	50.8		Employed	38	31.9
Female	58	49.2		Homemaker	36	30.3
**Age**	119		65.52 ± 7.82	Retired	44	37.0
**Level of education**				**BMI (kg/m**^**2**^**)**		
Low education	46	39.3		<25	9	8.0
Medium-level education	40	34.2		25-30	45	39.8
High education	31	26.5		30-35	33	29.2
				>35	26	23.0

^a^ The number of participants can differ between variables due to missing values.

Median levels of vigorous-intensity PA time increased (with 40.2% of all respondents actually increasing this behaviour), as did total walking time (with 62.4% having increased their walking time). Moderate-intensity PA time showed a borderline significant increase (p = 0.051), with 48.8% of the respondents having increased this behaviour at the second measurement. Total sitting time remained stable at the follow-up measurement (see Table 2).

**Table 2:** International physical activity questionnaire: 12-country reliability and validity

	**N**	**Median (Range) T0**	**Median (Range) T1 **^**a**^	**% Increased time spent in activity **^**b**^
**Total vigorous-intensity PA time** (min/day)	97	0.00 (0.00-48.85)	0.00 (0.00-161.14)*	40.2
**Total moderate-intensity PA time** (min/day)	86	12.85 (0.00-106.29)	17.14 (0.00-233.57)	48.8
**Total walking time** (min/day)	85	17.14 (0.00-60.00)	28.57 (0.00-180.00)*	62.4
**Total sitting time** (min/day)	76	360.00 (0.00-720.00)	360.00 (120.00-720.00)	42.1
**Outcome expectancies** (1–5)^c^	119	3.91 (3.00-5.00)		
**Attitude** (1–5)^**c**^	119	3.83 (3.00-5.00)		
**Self-efficacy** (1–5)^**c**^	119	4.00 (3.00-5.00)		
**Subjective Norm** (1–5)^**c**^	119	3.33 (1.00-5.00)		

^a^ Wilcoxon’s signed-rank test comparing scores at T0 and T1 * P < 0.001.

^b^ % of respondents of whom the Δ T1-T0 scores was > 0.

^c^ Score of 3 is a neutral score, a score > 3 is a positive score and a score < 3 is a negative score.

Linear regression analyses (Table 3) showed that a greater change in vigorous-intensity PA time between the baseline and follow-up measurements was negatively associated with education level and BMI at baseline. Highly educated respondents and those with a lower BMI had a smaller change in vigorous-intensity PA time between baseline and follow-up than low-educated respondents and respondents with a high BMI at baseline.

**Table 3:** Linear regression analyses predicting Δ vigorous-intensity PA time, Δ moderate-intensity PA time, Δ walking time and Δ sitting time

	**Δ vigorous-intensity PA time**				**Δ moderate-intensity PA time**				**Δ walking time**				**Δ sitting time**			
	**β**	**p**	**95% CI**		**β**	**P**	**95% CI**		**β**	**P**	**95% CI**		**β**	**p**	**95% CI**	
Gender^a^	0.15	0.54	−30.40;57.19		−0.32	0.17	−107.46;19.42		0.09	0.70	−37.29;55.38		−0.22	0.24	−193.71;49.63	
Age	−0.31	0.09	−3.96;0.29		−0.12	0.50	−4.12;2.06		**−0.45**	**0.01**	−5.34;-0.79		−0.13	0.35	−8.76;3.19	
*Education*^*b*^																
Low (reference)																
Medium	−0.28	0.10	−57.21;5.43		0.21	0.19	−15.83;76.86		−0.12	0.47	−45.97;21.55		0.13	0.34	−45.85;131.47	
High	**−0.60**	**<0.01**	−91.56;-18.09		−0.03	0.87	−57.22;48.80		−0.36	0.06	−76.38;2.23		0.14	0.38	−57.79;148.65	
*Occupation*^*c*^																
Employed (reference)																
Homemaker	−0.39	0.15	−89.95;13.69		0.33	0.20	−26.82;125.09		−0.27	0.27	−85.33;24.53		0.19	0.35	−76.95;211.54	
Retired	0.13	0.50	−24.17;49.11		0.02	0.94	−52.38;56.67		0.03	0.89	−36.86;42.27		0.15	0.33	−53.03;154.78	
Vigorous-Intensity PA t0	0.00	0.98	−0.99;1.01		**0.30**	**0.03**	0.15;3.05		−0.21	0.12	−1.91;0.24		−0.03	0.77	−3.22;2.40	
Moderate-Intensity PA t0	−0.07	0.65	−0.53;0.33		**−0.46**	**<0.01**	−1.70; -0.38		0.25	0.08	−0.06;0.92		0.05	0.71	−1.05;1.53	
Walking t0	0.16	0.24	−0.29;1.14		−0.11	0.43	−1.49;0.64		−0.09	0.47	−1.06;0.50		0.15	0.17	−0.63;3.44	
Total sitting time t0	0.06	0.70	−0.07;0.10		−0.16	0.29	−0.19;0.06		**0.30**	**0.04**	0.00;0.19		**−0.66**	**<0.01**	−0.89;-0.41	
BMI t0	**−0.35**	**0.01**	−5.57;-0.74		−0.07	0.59	−4.49;2.57		**−0.34**	**0.01**	−6.12;-0.78		**0.23**	**0.04**	0.37;14.40	
Motivation t0	0.05	0.75	−7.79;10.78		−0.04	0.77	−15.58;11.54		0.25	0.08	−1.26;19.67		0.03	0.80	−24.05;30.91	
*Cognitions*																
Outcome expectancies t0	−0.04	0.81	−42.58;33.27		−0.13	0.40	−79.34;32.10		−0.03	0.86	−44.14;37.03		**−0.26**	**0.04**	−221.22;-8.08	
Attitude t0	0.02	0.87	−35.23;41.38		−0.04	0.81	−66.94;52.88		−0.03	0.83	−45.96;37.27		0.12	0.32	−54.53;164.03	
Self-efficacy t0	−0.00	0.99	−34.38;33.78		−0.01	0.95	−50.51;47.24		−0.02	0.90	−38.34;33.94		−0.06	0.64	−117.38;72.44	
Subjective norm t0	0.15	0.32	−8.33;24.90		0.02	0.99	−24.53;24.93		0.10	0.47	−11.38;24.22		0.03	0.80	−40.71;52.77	
**R**^**2**^				0.34				0.41				0.43				0.61

Linear regression also showed that the change in moderate-intensity PA time was predicted by vigorous- and moderate-intensity PA time at baseline. More vigorous-intensity PA and less moderate-intensity PA time at baseline were associated with a greater change in moderate-intensity PA time.

The change in walking time was predicted by age, total sitting time at baseline and BMI at baseline. Younger respondents had a larger difference in walking time than older respondents. Furthermore, respondents with a high score for sitting time at baseline and those with a lower BMI at baseline had a greater change in walking time than those with low score for sitting time and a high BMI at baseline.

Finally, the difference in total sitting time was negatively associated with total sitting time and outcome expectancies at baseline, and positively associated with BMI at baseline (see Table 3). With the exception of the predictive value of baseline outcome expectancies for changes in sitting time, the socio-cognitive profile did not predict changes in PA behaviour or sitting time.

The sensitivity analyses testing the robustness of the results found in the regression analyses showed no major deviances from the original standardized regression coefficients, although most coefficients became non-significant due to the decreased statistical power because of the smaller sample size. Potential suppressor or mediation effects of PA behaviour in our multivariate equations were checked for by analysing bivariate correlations between the factors of the socio-cognitive profile and all outcomes. These Spearman correlations showed a range of −0.16 to 0.17 (mean 0.02), all non-significant. Regression analyses without the baseline PA behaviour and sitting time showed that there was no predictor effect of the socio-cognitive profile. Even the predictive effect of outcome expectancies on the sitting behaviour became non-significant in those analyses.

**Figure 1:**
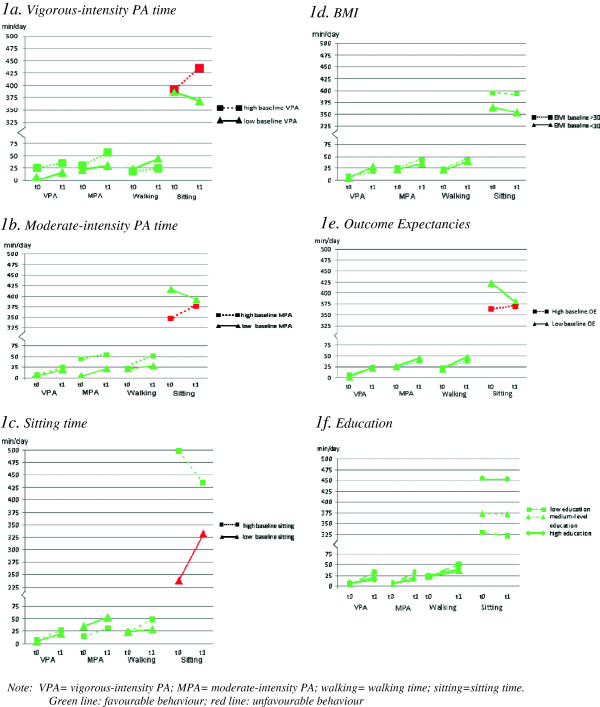
Graphic representation of changes in PA time, Sitting time, BMI, Outcome expectancies and education.

To assist the interpretation of the statistically significant regression results, figures were drawn using cut-offs based on the median baseline score for vigorous-intensity PA time (median score = 0), moderate-intensity PA time (median score = 12.85), sitting time (median score = 360) and outcome expectancies (median score = 3.91), as well as on BMI (non-obese vs obese) and education (low, intermediate, high). These figures showed that all subgroups had a tendency to show favourable changes two years after baseline (graphically represented by the green colour of the lines). However, some groups were found to have increased their sitting time: without exception, these were the groups with relatively favourable behavioural characteristics at baseline (i.e. high vigorous-intensity PA, high moderate-intensity PA, and low levels of sitting time). The subgroup with relatively high levels of outcome expectancies had also increased their sitting time. The groups that were distinguished on the basis of baseline moderate-intensity PA differed significantly in terms of the increase in moderate-intensity PA (p < .05). The groups with low and high scores for sitting time at baseline differed significantly in terms of the difference in sitting time (p < .001) and walking time (p < .05) (see Figure 1).

## Discussion

On average, respondents in this study were more physically active two years after the start of the intervention than they were at baseline, in terms of each level of intensity (walking and moderate- and vigorous-intensity PA, although the increase was non-significant for moderate-intensity PA). The baseline socio-cognitive profile did not predict intervention-induced changes in PA levels. The increase in moderate-intensity PA was best predicted by low baseline scores for moderate-intensity PA and high baseline scores for vigorous-intensity PA. The decrease in sitting time was best predicted by higher scores for sitting time and higher outcome expectancies, and by non-obese weight status at baseline.

The finding that the socio-cognitive profile of the respondents did not predict behavioural outcomes is in line with previous studies [24,25]. The limited predictive and mediating power of social cognitions in PA interventions has been the subject of some debate (e.g. [34,35]). Whereas social-cognitive theories see behaviour change as a linear, deterministic process, Resnicow and Vaughan [34] argued that decisions to initiate and maintain behaviour change are non-linear events. Behavioural changes may result from a surge of motivation or inspiration that is greater than the sum of its cognitive parts. This is something that happens beyond cognition, represented in statements such as ‘It just happened’, or ‘I just did it’. Our results appear to support this non-linear view of behaviour change. In this respect, our results indicate that GPs or other professionals should not be misled by patients’ expressions of high motivation or by perceived low levels of patient motivation or involvement. In fact, expressions of high motivation may reflect highly extrinsic levels of motivation, which are poor predictors of long-term behaviour maintenance [36], while relatively low levels of expressed self-efficacy may reflect a lack of optimistic bias and the inability to set realistic goals. Small units of positive belief structures may in the end lead to ‘quantum’ changes being made by apparently unmotivated individuals [34,37]. People may need to cross a ‘motivational threshold’ to start participating in combined lifestyle interventions, but we would advise intervention designers not to incorporate a high threshold of expressed motivation as a strict inclusion criterion.

Although respondents who scored favourably in terms of the PA measures at baseline increased their scores for PA measures, they also increased their sitting time. Studies have shown that respondents who increased their exercise level compensated this increase with more sedentary behaviour as a strategy to conserve energy [38,39]. It is therefore important to aim lifestyle interventions not only at increasing PA levels, but also at decreasing sitting time simultaneously. In addition, lifestyle interventions should give more attention to participants’ favourable behaviour (i.e. low baseline sitting time), rather than focusing only on behaviours that could be improved.

There was a positive association between baseline sitting time and the change in walking time. Our findings thus indicate that respondents with a high score for sitting time at baseline started with low-intensity activity such as walking. This is in agreement with the (stepped) goals of the BeweegKuur programme, in which participants are encouraged to start with low-threshold activities, such as walking and cycling.

The intervention we studied appeared to be suitable for people with a lower education, and our data shows that the intervention is indeed able to reach these people. Although the differences were non-significant, all outcomes appeared to develop more favourably among low-educated people than more highly educated people. A possible explanation why the BeweegKuur suits respondents with a lower education may lie in the referral by GPs. A study by Schmidt and colleagues [40] in the Netherlands showed that referral by GPs was an important motivation to participate, as this is a strong incentive and provides a legitimate reason for starting to exercise. The fact that the BeweegKuur was free of charge for the participants has probably resulted in the relatively large low-SES sample. In addition, the frequent contacts with the LSA, the use of Motivational Interviewing techniques and the tailored BeweegKuur programme may have made the intervention suitable for people with a lower education.

Strengths of the current study include the relatively large sample of diabetic or pre-diabetic patients, the longitudinal design, the long-term follow-up and the use of a theory-based research framework of social-cognitive factors. A limitation of the present study was its non-controlled design. This non-controlled design influences the validity of the results, because it was not possible to ascertain whether the changes in outcome variables were a consequence of the BeweegKuur, or the result of other causes. It is recommended to use a controlled design in future research. Another limitation was the use of self-report measures. The use of self-report measures can lead to socially desirable answers and thus to underestimation (as regards weight) and overestimation (as regards PA time). The best way to avoid this in future studies would be to combine self-reported measurements with objective measurements, such as accelerometers to assess activity levels. All respondents in our study had voluntarily agreed to participate in the intervention, which may explain the high average level of motivation. Another limitation of this study was that no process parameters regarding the intervention (e.g. attendance to or accomplishment of the programme) were taken into account. Also, there was no measurement immediately after the programme, and we have no information about the respondents’ behaviour during the year after the programme. Another point of concern is the high drop-out rate. This could be explained by the fact that participants were approached and reached by the LSA. Questionnaires were sent to the LSA, who had to provide them to the participants. It was impossible for us to detect if all participants in the BeweegKuur were reached with this method. It is possible that some participants did not receive the questionnaire or that they received it at a different time than the researchers indicated. The high drop-out rate along with the different characteristics of the dropouts and completers, could have led to a selection bias. Therefore, the results should be interpreted with caution. Additional research would be needed to examine whether our results would be similar in other risk groups and in other countries.

## Conclusions

To conclude, two years after the start of the intervention, respondents appeared to spend more time being physically active. Respondents who had relatively high levels of physical activity at the start of the intervention, reported to have increased their total sitting time, while respondents with relatively low levels of physical activity at the start reported a decreased sitting time. The intervention appeared to be suitable for people with a low-education level, but the results should be interpreted in view of the limitations of the study such as the non-controlled design, self-reported outcomes and selective drop-out of participants. Interventions for this specific target group may need to put more emphasis on the prevention of increased sitting time. The finding that the socio-cognitive profile did not predict behaviour change may underline the proposition that decisions to initiate and maintain PA behaviour change are to a large extend non-linear events. Acknowledging the possible non-linearity of the relationship between socio-cognitive determinants and behaviour change will help our understanding of this complex and dynamic process. The findings may perhaps also be generalized to other high risk and patient populations, although caution is warranted.

## Competing interests

The authors declare that they have no competing interests.

## Authors’ contributions

JH conducted the study and wrote the first draft of the paper. All authors (JH, JG, FvB, NdV and SK) have read and contributed to earlier versions of the manuscript and have approved the final manuscript.
